# A Numbering System for MFS Transporter Proteins

**DOI:** 10.3389/fmolb.2016.00021

**Published:** 2016-06-02

**Authors:** Joanna Lee, Zara A. Sands, Philip C. Biggin

**Affiliations:** ^1^Department of Biochemistry, University of OxfordOxford, UK; ^2^UCB Pharma S.A.Braine-l'Alleud, Belgium

**Keywords:** homology modeling, LacY, alternating access, transport, transmembrane

## Abstract

The Major Facilitator Superfamily (MFS) is one of the largest classes of secondary active transporters and is widely expressed in many domains of life. It is characterized by a common 12-transmembrane helix motif that allows the selective transport of a vast range of diverse substrates across the membrane. MFS transporters play a central role in many physiological processes and are increasingly recognized as potential drug targets. Despite intensive efforts, there are still only a handful of crystal structures and therefore homology modeling is likely to be a necessary process for providing models to interpret experiments for many years to come. However, the diversity of sequences and the multiple conformational states these proteins can exist in makes the process significantly more complicated, especially for sequences for which there is very little sequence identity to known templates. Inspired by the approach adopted many years ago for GPCRs, we have analyzed the large number of MFS sequences now available alongside the current structural information to propose a series of conserved contact points that can provide additional guidance for the homology modeling process. To enable cross-comparison across MFS models we also present a numbering scheme that can be used to provide a point of reference within each of the 12 transmembrane regions.

## Introduction

The Major Facilitator Superfamily (MFS) is the largest known superfamily of secondary transporters (Marger and Saier, 1993) expanding in recent years to contain 74 distinct families according to the Transporter Classification Database (TCDB; Saier et al., 2014). These transporters operate by uniport, symport, or antiport mechanisms that take advantage of the electrochemical gradient of the co-transported ion (in the case of symport or antiport) or the concentration of the ligand to instigate the transport cycle (Pao et al., 1998). MFS proteins transport a huge variety of ligands including monosaccharides, drugs, enzyme cofactors, peptides, oligosaccharides, nucleotides, iron chelates, and inorganic anions and cations (Burckhardt and Wolff, 2000; Saier and Paulsen, 2001; Guan and Kaback, 2006; Newstead et al., 2011).

**Figure 1:**
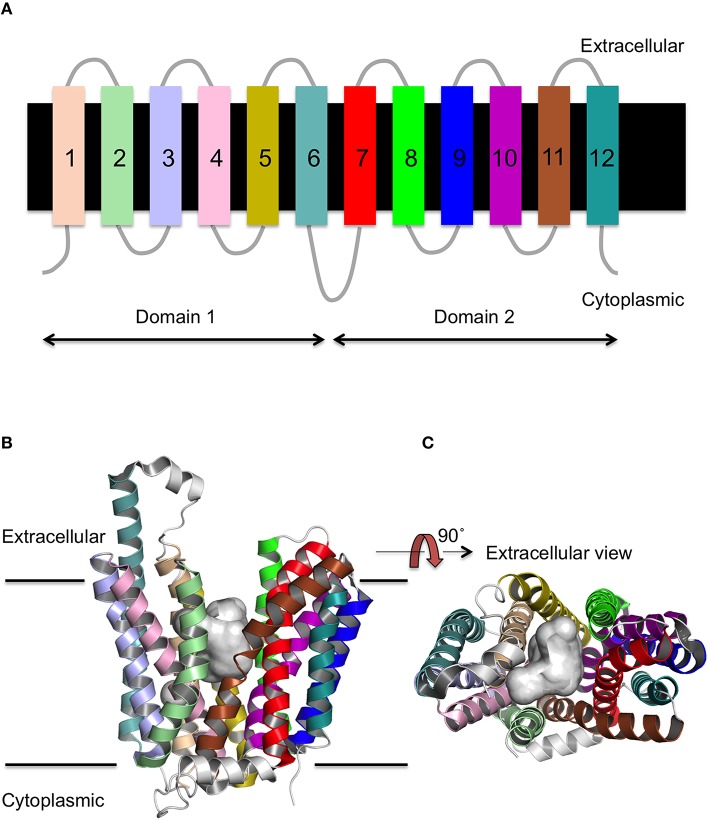
**Secondary and tertiary structure of MFS proteins. (A)** The topology of the 12 TM helices. **(B)** The MFS fold as viewed from the side of the membrane, and **(C)** rotated through 90 as exemplified by FucP. The conserved 12 TM α-helix fold is arranged into two 6 TMH bundles (domains 1 and 2) and a cavity exists (gray surface) at the interface between them that is accessible to either the cytoplasm or extracellular region depending on the conformational state.

MFS proteins have a conserved 12 transmembrane (TM) α-helix fold (Figure 1A; Reddy et al., 2012) that is comprised of two 6-TM helix bundles that are related by a pseudo two-fold axis of symmetry. The presence of both domains is thought to be functionally important for the transport mechanism, as the ligand binds to the central TM cavity at the interface between the two domains (Pazdernik et al., 1997; Figures 1B,C). The 12-TM topology appears to be the core fold, though the presence of additional helices is sometimes observed as for example seen in peptide transporters (Newstead et al., 2011). The arrangement of the helices within the distinct conformations of the X-ray crystal structures suggests that transmembrane helices (TMH)s 1, 4, 7, and 10 line the path of the substrate through the transporter, whilst TMHs 2, 5, 8, and 11 mediate the interface between the two domains (Yan, 2015).

There is some controversy as to the evolution of the six TM domains, which may have arisen through nucleotide duplication in the gene for either a 2-TM or 3-TM helix segment. The 3-TM helix repeat motif was proposed by Radestock et al. (Radestock and Forrest, 2011), who showed that an outward facing model of LacY generated via their approach had a backbone atom root mean squared deviation (RMSD) of 3.2 Å (over the 12 TM helices) when fitted to the crystal structure of FucP. Furthermore, alignments using this approach can improve the apparent sequence similarity when compared to alignment of the full sequences. This hypothesis has been extended in recent years (Keller et al., 2014), culminating in a mix-and-match theory (Madej, 2015).

However, this triple helix motif hypothesis is disputed by (Västermark and Saier, 2014), who direct attention to the conserved MFS motifs in the cytoplasmic loops between TMHs 2–3 and 8–9. These loop motifs align best when using the entire protein sequence, rather than the triple helix motifs described by Radestock et al. (Radestock and Forrest, 2011). In addition to the motif on these cytoplasmic loops (GxlaDrxGR; Paulsen et al., 1996), there is a further motif present on TMH 4 that is comprised of at least one conserved glycine residue (Pascual et al., 2008).

MFS transporters are thought to operate via an alternating access mechanism (Kasho et al., 2006; Eraly, 2008; Kaback et al., 2011; Smirnova et al., 2011, 2014). In this mechanism, the MFS protein undergoes a series of conformational changes to allow the passage of a ligand from the extracellular solution into the cytoplasm or vice versa. In MFS proteins, the most studied region is that of the ligand-binding site that is situated between the two domains in the central TM cavity. The paradigm has been the lactose permease (LacY) that operates by a symport mechanism and transports both sugar (predominantly lactose or galactose) and H^+^ in the same direction across the membrane (Guan and Kaback, 2006; Madej et al., 2014). The mechanism of sugar transport in LacY has been extensively investigated and the key residues involved in either lactose binding or H^+^ translocation are well-characterized. The ligand-binding site is formed by E269, E325, H322, R302, and Y236, which are predominantly charged residues at neutral pH. Thus, the LacY central cavity is fairly hydrophilic in character, at least at the point of ligand binding and transport is mediated by TMHs 5, 7, 8, 10, and 11. The positions of residues involved in substrate transport are similar in other MFS proteins (Madej and Kaback, 2013; Madej et al., 2013).

The role of the loop regions in MFS proteins is less well-explored. However, Masureel et al. (2014) recently determined that key, titratable residues, were necessary in the loop regions of LmrP in order for conformational change to proceed. Double electron electron resonance (DEER) experiments have defined the distance between helix pairs in LmrP and the effect of pH on the relative distances was measured. The relative abundance of each distance correlated to the conformation of the protein. It was shown that at pH 5, LmrP would reside in the outward-closed state, but at pH 8 an outward-open state was preferred. Like LacY, LmrP is thought to operate as a drug/H^+^ symporter and these results suggest that the pH conditions of the environment can affect the movement of substrate by shifting the conformational equilibrium of the protein.

There are 104 identified human MFS proteins in the Genomic Transport database (Ren et al., 2004) and most are classified as solute carriers (SLC). Many of these are of interest from a pharmaceutical point of view (Lin et al., 2015). For example, the SLC18 protein is a potential marker for Parkinson's disease (Giboureau et al., 2010) and glutamate transporters are being investigated as drug targets for neuropsychiatric and neurodegenerative diseases (Hinoi et al., 2005). Probenecid is an SLC22 (organic anion transporter) inhibitor whose co-administration is used in cases of nephrotoxicity (Devineni et al., 2015). Furthermore, SV2A is the drug target for levetiracetam, a successful anti-epileptic drug (Klitgaard et al., 1998; Löscher et al., 1998; Lynch et al., 2004).

Although, there are an increasing number of X-ray crystal structures for MFS proteins (Yan, 2016), there are still many examples where there is no structural information. In that case one has to use homology modeling (Kasho et al., 2006) to provide a working model. This can be a powerful tool for drug discovery, but the quality of the model is important in order to have confidence in screening for potential compounds in the binding site. The biggest factor in determining the quality of a model is the sequence alignment. For MFS proteins, sequence identities are very low between families and are said to sit within the so-called “twilight zone” (Rost, 1999). As a consequence achieving a reliable alignment is not trivial and even hidden Markov models (HMMs) struggle in this regard. Nevertheless, good starting models can provide valuable information for the design of experimental strategies, such as site-directed mutagenesis, in order to understand the molecular basis of solute (and co-solute) transport.

One strategy to improve confidence in the alignment is to focus on “anchor” points—residues that are highly conserved or have additional evidence that they are likely to be located at important regions within the protein. Identification of such points can in turn also help to provide a means to compare structures or models from diverse sequences with the same fold. Such an approach was implemented by Ballesteros and Weinstein (and recently updated; Isberg et al., 2015) with respect to GPCRs (see Table S1) and has been shown to aid homology modeling of GPCRs in drug design programs (Zhou et al., 1994; Almaula et al., 1996; Javitch et al., 1998; Pascual et al., 2008).

The conservation of residues according to distinct physicochemical properties can be used in multiple sequence alignment (MSA) analysis to guide the position of contacts between helices. Small or hydrophobic residues predominantly mediate these contacts, but hydroxyl groups or aromatic residues can also be present at helix–helix contact points. Buried hydrophobic residues within the TM region that are not solvent accessible tend to be the most highly conserved regions of TM proteins (Eyre et al., 2004). These properties can be used to predict the contacts between TM helices in MFS proteins. Indeed, this approach was utilized for GPCRs to show a network of contacts that are predominantly between TMHs 1–2, 1–7, 3–4, 3–5, and 6–7 (Venkatakrishnan et al., 2013). Defining similar contacts within MFS proteins will help provide insights into which intrahelical interactions are important and which change during the transport cycle.

The vast number of sequences now available, thanks to modern sequencing techniques, allows us to generate MSA for proteins with high sequence similarity to MFS transporters for which the crystal structure is known. These alignments were then compared with close contacts within all the current MFS structures to identify conserved interaction points and thus develop a set of rules to aid homology modeling of MFS transporters.

## Methods

### Multiple sequence alignment

Since MFS proteins have low sequence identity with each other (Table 1), a MSA of the whole superfamily was not feasible for determining the conservation of residues that relate to structure (Tramontano, 1998). It is highly unlikely that the alignment would be optimal. It was difficult to define at what point sequences become sufficiently similar to allow us to infer structural information (Rost, 1999). We initially explored the use of Pfam (Finn et al., 2014), but this turned out to be too diverse in terms of clan members, as indeed has been discussed when compared to the Transporter Classification Database (Chiang et al., 2015).

**Table 1:** **The sequence identity (%) between 10 MFS proteins for which there is an X-ray crystal structure**.

**Protein**	**LacY (1PV6)**	**FucP (3O7P)**	**XylE (4GBY)**	**GlpT (1PW4)**	**PepT (2XUT)**	**EmrD (3GFP)**	**YajR (3WDO)**	**PiPT (4J05)**	**NarK (4JR9)**	**GLUT1 (4PYP)**
LacY (1PV6)										
FucP (3O7P)	19									
XylE (4GBY)	22	22								
GlpT (1PW4)	21	19	23							
PepT (2XUT)	23	24	19	24						
EmrD (3GFP)	21	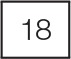	20	21	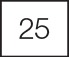					
YajR (3WDO)	22	21	25	20	23	22				
PiPT (4J05)	22	21	24	19	22	23	22			
NarK (4JR9)	21	22	22	23	23	24	23	21		
GLUT1 (4PYP)	21	23	29	21	20	22	20	22	24	

The percentage identity was computed from CLUSTAL Omega pairwise alignments and they range from 18 to 25.

Given that we now have many more sequences available, we thus decided to use the UniRef50 clusters (Suzek et al., 2007) for each protein solved by X-ray crystallography (Table 2). These are sequences that have at least 50% identity and we took the view that as long as the identity was above 50%, the alignment quality would be sufficient (Baker and Sali, 2001) to provide useful alignments. MSAs were then constructed using the MUSCLE algorithm (Edgar, 2004a) incorporated into a python script.

**Table 2:** **The MFS proteins used for determining key residues that are conserved across the superfamily in this work**.

**Protein**	**MFS family**	**UniRef50/UniProt ID**	**Substrate**	**Transport direction**	**Species**
FucP	MFS_1	P11551	Fucose/H^+^ symport	Into cell	*E. coli*
GlpT	MFS_1	P08194	Glycerol-3P/Pi antiport	Into cell	*E. coli*
EmrD	DHA12	P31442	Multidrug/H^+^ antiport	Out of cell	*E. coli*
YajR	DHA12	P77726	Multidrug/H^+^ antiport	Out of cell	*E. coli*
LacY	lacy_symport	P02920	Galactose/H^+^ symport	Into cell	*E. coli*
PepT	POT	Q8EKT7	Peptide/H^+^ symport	Into cell	*Streptococcus thermophilus*
YgbH	POT	P75742	Peptide/H^+^ symport	Into cell	*E. coli*
GlcPse		Q8CQA7	Glucose/H^+^ symport	Into cell	*Staphylococcus epidermis*
gkPOT	POT	Q5KYD1	Peptide/H^+^ symport	Into cell	*Geobacillus kaustophilus*
GLUT1	SP	P11166	Glucose uniport	Into cell	*Homo sapiens*
PiPT	SP	A8N031	Phosphate/H^+^ symport	Into cell	*Piriformospora indica*
NRT1.1	NRT1/PTR	Q05085	Nitrite/H^+^ symport	Into cell	*Arabidopsis thaliana*
NarU	NNP	P37758	Nitrate/nitrite symport	Into cell	*E. coli*
XylE	SP	P0AGF4	Xylose/H^+^ symport	Into cell	*E. coli*
MelB	Glycoside-pentoside-hexuronide: cation symporter	P30878	Melibose/Na^+^ symport	Into cell	*Salmonella typhimurium*

The cluster containing each protein sequence in UniRef50 was downloaded and aligned using MUSCLE (Edgar, 2004b).

### Analysis of MSA

Conservation at each site was determined using an in-house R script (available upon request) according to the chemical groupings of the amino acids: hydrophobic (M, A, V, I, L, C, Y, F, W), polar (S, T, N, Q), positive charge (R, H, K), negative charge (D, E), aromatic (W, F, Y), glycine (G), or proline (P). Data were visually represented using the heatmap.2 package in R. This analysis was applied to the MFS proteins listed in Table 2.

### Contact prediction and analysis

The contacts between helices were defined by distance (side chain atoms within 7 Å of the adjacent helix backbone atoms). The VMD package (Humphrey et al., 1996) was used to find all contacts within crystal structures based on this definition. We analyzed 44 MFS crystal structures for contacts between TMH pairs (see Figures S1–S3). The resolution of the crystal structures ranges from 1.9 Å (4IKV:POT peptide transporter) to 4.2 Å (4JA4: xylose transporter). Although, higher resolution structures may define side-chain positions more accurately we do not anticipate this to dramatically influence the results of the contact analysis. Heat maps were constructed which showed the presence or absence of the particular residue type contact for all potential helix–helix interactions. We then assessed the location within the bilayer of conserved positions according to the OPM (Orientations of Proteins in Membranes) database (Lomize et al., 2006).

## Results and dicussion

### MSA analysis

Many of the previous observations regarding conserved sites (Pao et al., 1998) are reinforced by the MSA analysis here and we will not go into detail, but just emphasize some of the key points. Two of the conserved sites highlighted by the MSA analysis are the glycine residues on TMH 4 and its symmetrically equivalent helix in domain 2, TMH 10. In every crystal structure these glycine residues are conserved. There is predominantly only one glycine in TMH 10, but there are between one and four conserved glycine residues in TMH 4. A similar pattern continues for all conserved sites common to the helices in domains 1 and 2. This implies that even if the domains arose from an evolutionary repeat (Reddy et al., 2012) it was very distant in time and so the domains have evolved to have considerable dissimilarity between them. Presumably this reflects the need for certain MFS proteins to become specialized and enable them to bind different substrates. Intragenic duplication of two TM domains and subsequent evolution has been argued as the most likely explanation for the variation observed within the MFS proteins (Reddy et al., 2012). Similarly, the conserved aromatic residue at the center of TMH 7 does not exist as a conserved residue in TMH 1. In LacY, Y236 (on TMH 7) is known to be functionally important and so it could be that there is functional evolutionary pressure that maintains the chemical nature of the residue at this position. Mutation of this residue has previously been shown to impair transport (Guan and Kaback, 2006).

There are several positions that reflect the expected symmetry of the domains. A glycine at the center of TMH 5 is also present in TMH 11 and the positive residue in loop 2–3 is present in loop 8–9. The positive residue in loop 2–3 is part of a conserved motif known to exist in the MFS proteins (Pao et al., 1998) and the positive residue of that motif is mirrored in domain 2 (loop 8–9). Positively charged residues also exist in loops 4–5 and 10–11, indicating that MFS transporters conform to the positive-inside rule (von Heijne, 1992). A negative charge at the end of TMH 6 is also mirrored in TMH 12. However, its position was not always conserved at the level of the lipid head groups, but instead in loop 6–7 or near the N-terminus.

**Figure 2:**
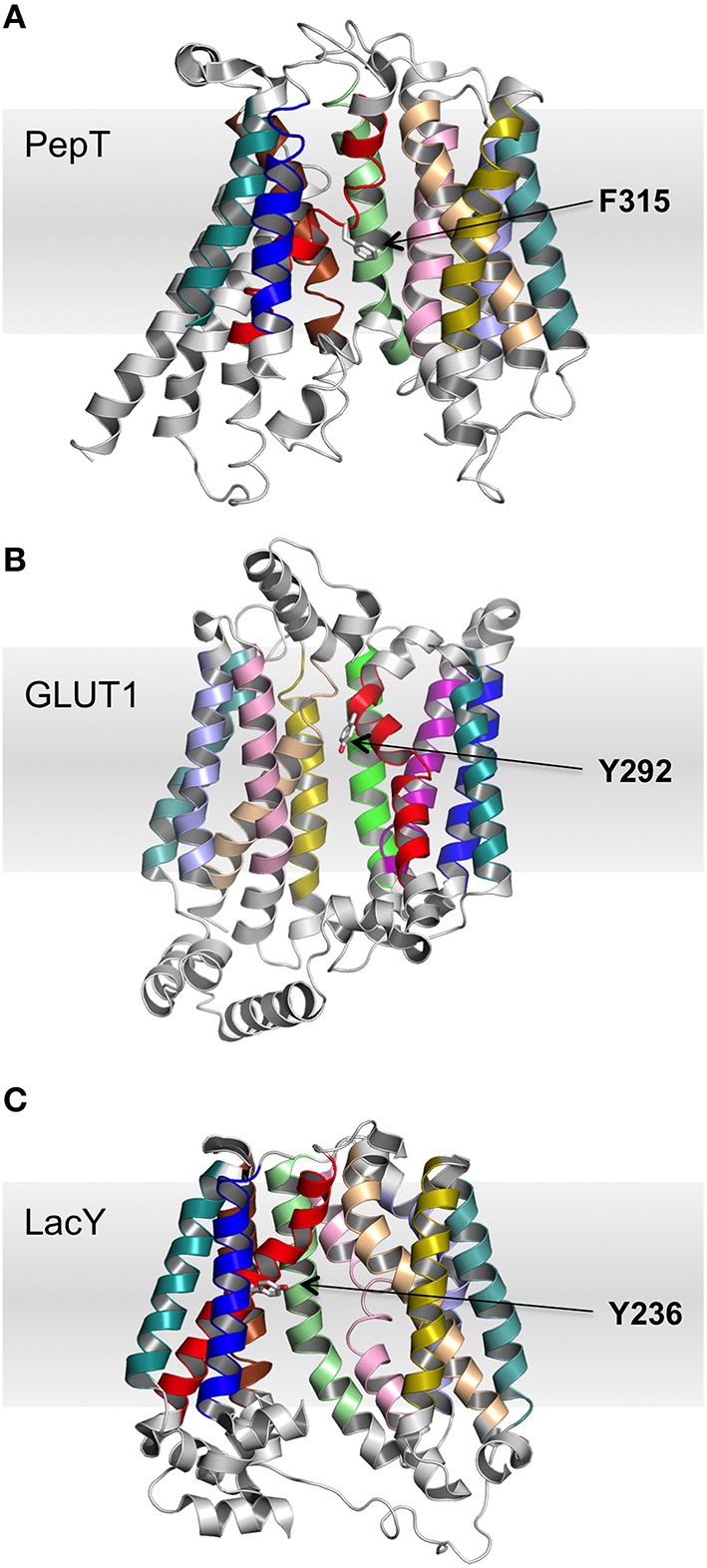
**The aromatic side chain on TMH 7. (A)** PepT (occluded), **(B)** GLUT1 (outward), and **(C)** LacY (inward; PDB IDs 2XUT, 2PYP, and 1PV6, respectively). In both proteins, the most conserved aromatic residue points into the central TM cavity between the two domains, but the absolute position with respect to the membrane is variable. For clarity, helices 8 and 10 are omitted from **(A)** and helices 2 and 11 are omitted from **(B)**.

There are some apparent anomalies. In particular, EmrD does not have the aromatic residue in TMH 7 or the positive charge in loop 10–11. With regards to the conserved aromatic residue on TMH 7, in some proteins there is more than one conserved aromatic residue. In order to identify which is the functionally or structurally relevant residue, the position of these aromatic residues was related to the percentage conservation. Those that are most conserved are also those pointing into the central cavity, as seen in PepT (2XUT) and GLUT1 (4PYP). These residues have similar orientations to the functionally important residue in LacY (Y236; Figure 2). A similar issue arises with TMHs 4, 5, 10, and 11, which may contain more than one conserved glycine and so these positions cannot always be used in isolation to aid homology modeling.

### To what extent are contacts conserved?

**Figure 3:**
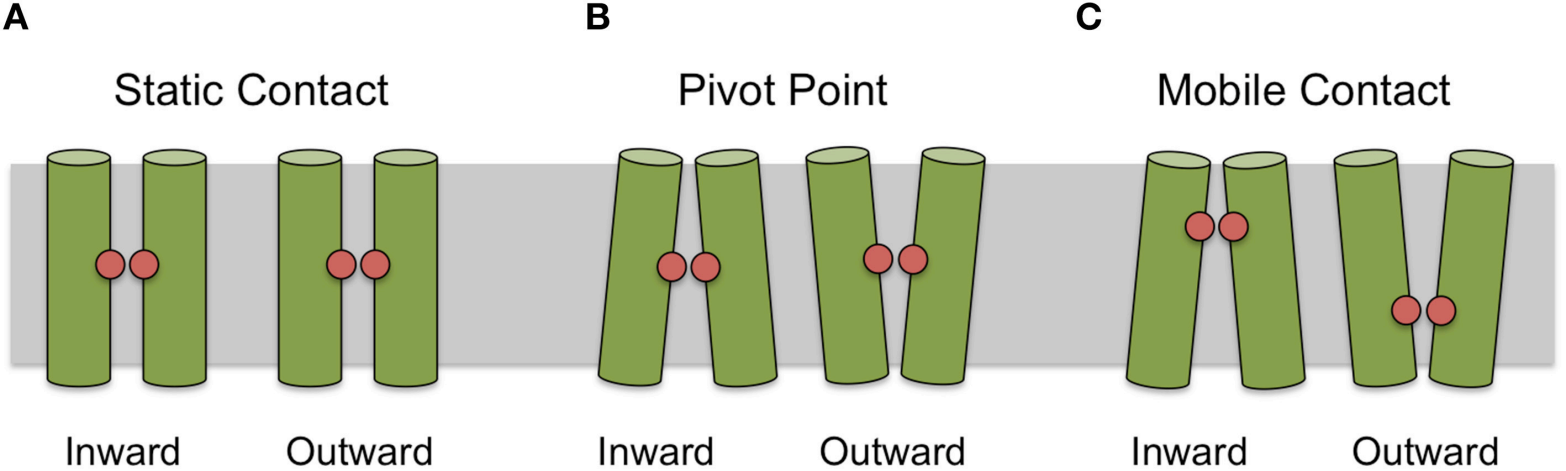
**Classification of contact types**. Contacts can be classified as static **(A)** where there is apparent different between conformational states, as pivot points **(B)** where the contact remains static but the angle of the helices making the interaction changes between conformational state or mobile **(C)** where the position of the most conserved contact between helices appears to depend on conformational state.

For some helices it was not possible to identify a uniquely conserved position from sequence analysis alone. Therefore, we manually examined the 44 crystal structures for helix–helix contacts (see Methods Section). The resulting contacts can be broadly classed into three groups: (1) those that mediate the packing of helices and where the relative orientations of the helices is invariant; (2) those where the contact is conserved but the orientation of the helices changes according to different conformational states (we refer to these as pivot points); and (3) those whose position exhibits some apparent dependence on the conformational state of the protein. For example a particular contact may only be conserved in one particular conformational state (such as the inward open for example) and a different contact between those two helices may be present in an alternative conformational state. Figure 3 summarizes these different classes of contacts schematically.

**Figure 4:**
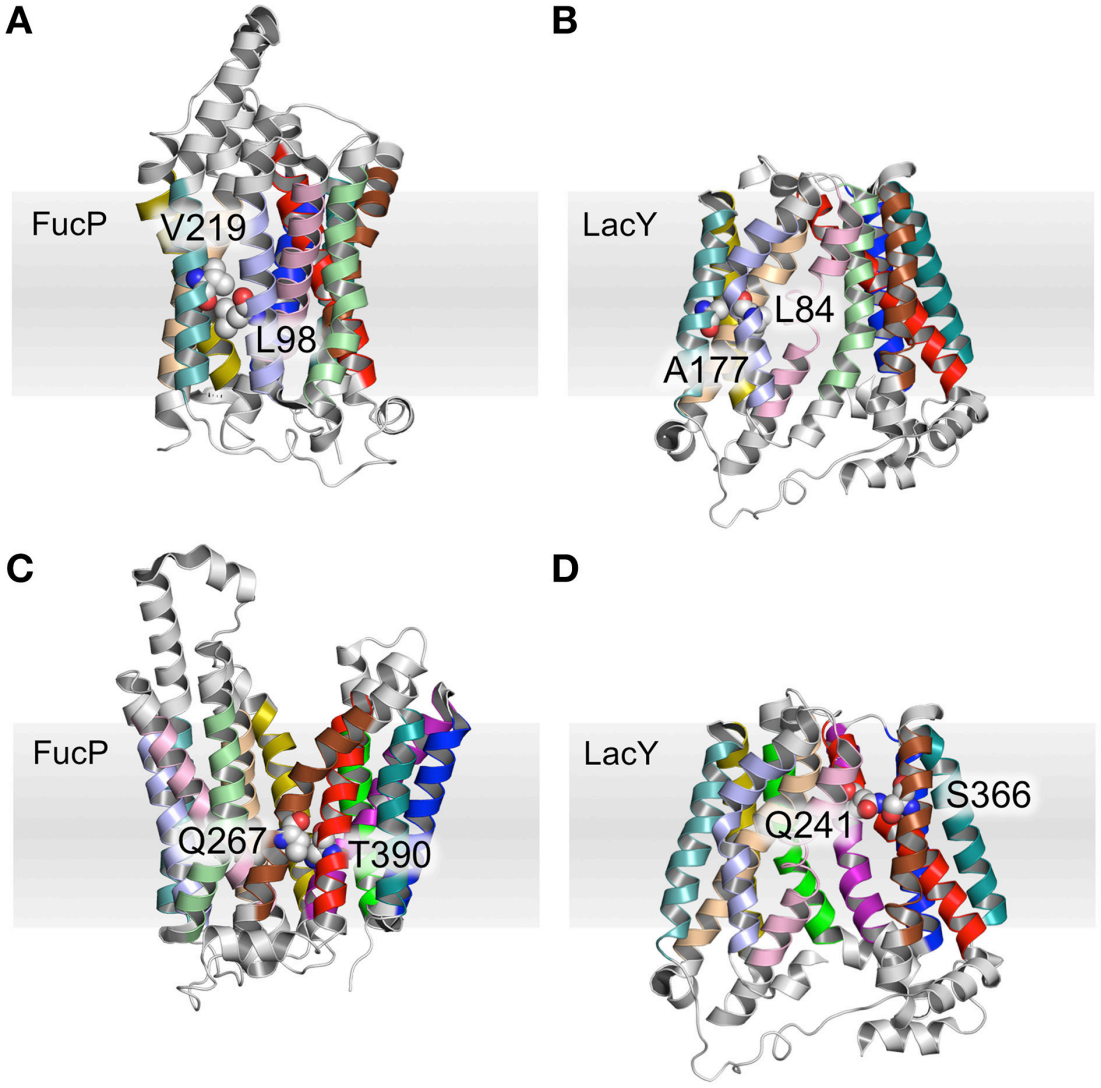
**Pivot point contact and variable contacts**. An example of an equivalent pivot point contact between TMH 3 and TMH 6 for FucP **(A)** between L98 (helix 3) and V219 (helix 6) and LacY **(B)** between L84 (helix 3) and A177 (helix 6). An example mobile contact between TMHs 7 and 11 is similarly depicted for FucP between Q267 (helix 7) and T390 (helix 11) **(C)** and for LacY between Q241 (helix 7) and S366 (helix 11) **(D)**. Helix 2 has been removed for clarity in **(D)**.

Table 3 summarizes the contacts conserved across all 44 structures. Helix 2 makes a contact with helices 1 and 11 at the cytoplasmic side of the membrane that is conserved regardless of conformational state and thus may be particularly useful in improving alignments in the future. Four scaffold residues (small hydrophobic) have previously been described in the literature (Doki et al., 2013; Yaffe et al., 2013). The contacts of these residues also remain unchanged across the conformations and they are therefore classified as pivot points. From our analysis, it is apparent that these mediate contacts between TMHs 3–6 and 9–12 (Table 3, Figures 4A,B).

**Table 3:** **Analysis of the position of the most conserved contacts between helices (summarized by a series of heat maps in **Figure 5** and Figures S1–S3)**.

**Residue involved in contact**	**Helix–Helix**	**Description**
STNQ	1–2	Keeps periplasmic end of helices in contact regardless of state
STNQ	2–11	Keeps periplasmic end of helices in contact regardless of state
STNQ	5–8	In the center of the TM region, and state dependent
STNQ	7–11	In the center of the TM region, and state dependent
Small hydrophobic	1–5	Contacts are at a pivot point in the helices, with each conformation having a different tilt to bilayer normal
Small hydrophobic	1–6	The contact is in the middle of the helix and TMH 1 has different tilts for each state
Small hydrophobic	2–4	The contact is in the middle of the helices and small changes in tilt
Small hydrophobic	2–11	The contact is along length of helices in occluded and inward structure but only at cytoplasmic end in outward structure
Small hydrophobic	3–4	Mediates packing
Small hydrophobic	3–6	Mediates packing
Small hydrophobic	5–8	The contacts are in the center of TM region
Small hydrophobic	7–11	In cytoplasmic half of the helices, outward state TMH 7 bends away from TMH 11 at periplasmic end to occluded and inward
Small hydrophobic	8–10	The contacts are along the helices—mediates packing
Small hydrophobic	9–10	The contacts are along the helices—mediates packing
Small hydrophobic	9–12	The contacts are along the helices—mediates packing, all three states have straight helices
Large hydrophobic	2–4	The position of the contact is in the center of the TM region, and the helices rock around that central point
Large hydrophobic	3–6	At the periplasmic end of the TM region. Inward structure has a further contact at cytoplasmic end of the TM region
Large hydrophobic	8–10	The contacts are in the center of the TM region, with a small difference in TMH 10 tilt between the conformations
Glycine	1–5	The contact is in the center of the helices, and is a pivot point for the helices which have different tilts in each state
Glycine	2–4	The contact is in the center of the helices, not much difference in tilt implying a packing motif
Glycine	9–10	The contacts are along the length of the helices, indicating packing mediators

The analysis depicts either static contact points (green) that do not change between different conformational states; contacts that move according to conformational state (blue), and contacts that constitute distinct pivot points (purple) around which the rest of the protein moves.

A further 12 interactions were identified that provide either pivot or moving (according to the conformational states) helix–helix contacts (Table 3, and exemplified in Figures 4C,D). The two inter-domain contacts are mediated by interactions between TMHs 2–11 and 5–8. It is expected that these would have varied contacts according to the conformational state. This is the case for TMHs 5–8 where the helices move around two pivot point contacts formed by polar and small hydrophobic residues.

However, the nature of interactions between TMHs 2–11 is less clear-cut. Our analysis suggests that the small hydrophobic contact points are conformationally dependent, but that the polar contacts would be classified as invariant. We note here that the data is skewed toward occluded and inward open conformations (only two structures have on outward open state: FucP and GLUT1) and therefore caution should be applied in the interpretation here.

Certainly the structures so far suggest that the largest conformational change is from outward to occluded, rather than from occluded to inward. If this is a genuine trend, this implies that the motion of the TMHs 2, 5, 8, and 11 is less important to the transport cycle than helices: 1, 4, 7, and 10. Indeed, TMHs 7 and 10 in the PepT_So_ transporter have previously been highlighted as being dynamic (Newstead et al., 2011). In addition Doki et al. (2013) reported TMH 4 as being dynamic in the GkPOT transporter.

All the remaining conserved contacts (Table 3) are intra-domain (i.e., within TMHs 1–6 or within TMHs 7–12) and reflect the movements that the helices undergo in the transport cycle. In domain 1, there are two hydrophobic contact points made by TMH 1: TMHs 1–5 and TMHs 1–6. The former acts as a pivot point, but the latter shows variation according to the conformational state.

For the equivalent TMH in domain 2, (TMH 7), there is no 7–12 contact point (at least not as conserved as the 1–6 contact), but there are two TMH 7–11 contacts mediated by polar and small hydrophobic residues (Table 3). Both of these contact points are conformationally dependent though.

In terms of the conformationally dependent contacts, those formed by TMH 1 or THM 7 exhibit the largest variations. A prediction that arises from the triple-helix repeat model (Radestock and Forrest, 2011) is that a similar variation would occur for TMHs 4 and 10, but this was not evident from our contact analysis.

There are nine contacts that we have assigned as static (Table 3). That is to say these contacts remain in the same position, regardless of the conformational state of the protein. They are predominantly between TMHs 3 and 4, TMHs 3 and 6, TMHs 8 and 10, TMHs 9 and 10, and TMHs 9 and 12. The invariant nature of these contacts, despite differing conformations, renders them as useful anchor points for supporting MFS homology modeling. Since the contacts remain in the same position in all MFS proteins, they serve as a guide to predict the orientation of helices in MFS targets through sequence alignment.

An important caveat to note concerns the way in which the helices move in the transport cycle. The theory is that the domains could move through a rigid body rotation (Shi, 2013) and therefore there ought not to be too many movements in the relative position of helices within domains. Conversely, the inter-domain contacts should move as the domains rock against each other. However, our analysis shows that small movements occur in the contacts between TMHs 2 and 11 or between TMHs 5 and 8. This suggests that there are some independent rearrangements of the helices on top of the predicted rigid body movement of domains.

**Figure 5:**
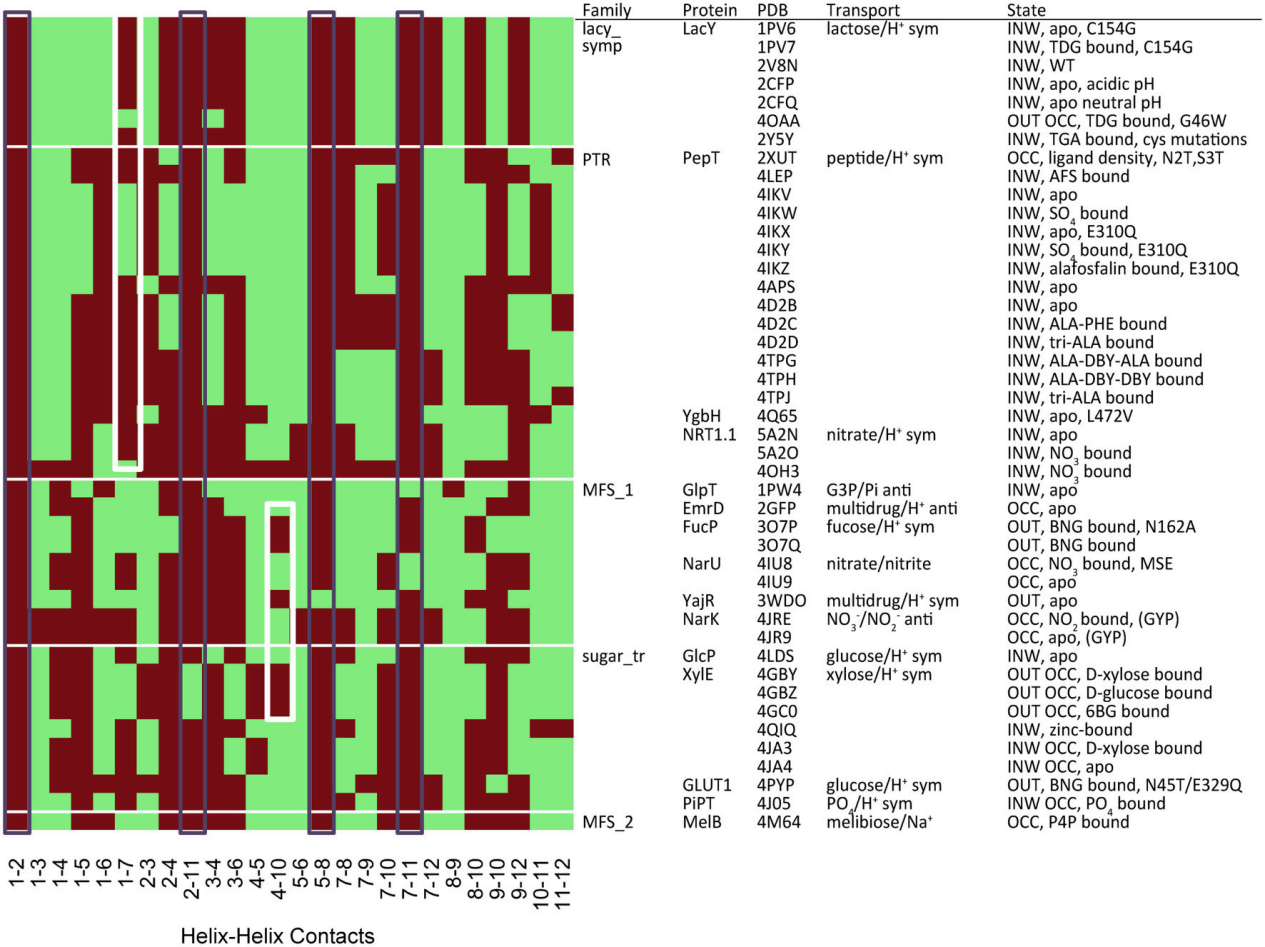
**Contact map for polar residues (S, T, N, Q)**. The black boxes show the most conserved contacts, whilst the white boxes indicate the position of contacts that describe the degree of closure of the cavity at both the cytoplasmic (TMH 4–10) and extracellular (TMH 1–7) ends. Heat maps analysis for other residue groupings can be found in the Figures S2–S4.

There are two metrics that describe the degree to which either side of the transporter is open. When TMHs 1 and 7 interact, the periplasmic side of the TM cavity is closed and when TMHs 4 and 10 interact, the cytoplasmic side is closed. Therefore, we used these residues to describe the degree of closure of either side of the TM cavity. It is possible to see such contacts in the heat-map of polar residue contacts (Figure 5, white boxes), where the TMH 4–10 contact is present in the outward open X-ray crystal structures and the TMH 1–7 contact is present in the inward open structures. Heat maps for other contact types are shown in Figures S1–S3.

### A suggested numbering system to help MFS modeling

Considering the identification of conserved residues alongside the identification of conserved contact points, we postulated whether it would be possible to use this information to provide common reference points within the MFS topology that could be useful for guiding homology modeling of these proteins. Furthermore, it should facilitate structural comparison across the entire family. Inspired by the Ballesteros and Weinstein approach to GPCRs (Table S1), we thus devised a set of rules that allow cross-family conserved sites to be compared. The most conserved positions are defined as x.0, where x is the TMH (Marger and Saier, 1993; Pazdernik et al., 1997; Pao et al., 1998; Burckhardt and Wolff, 2000; Saier and Paulsen, 2001; Guan and Kaback, 2006; Newstead et al., 2011; Radestock and Forrest, 2011; Reddy et al., 2012; Keller et al., 2014; Saier et al., 2014; Yan, 2015). In cases where conserved residues across the superfamily do not exist (i.e., not present in Table 3, Tables S2–S15), then the conserved contact points are used to define position 0.

#### Rules for numbering

1. Residues that are conserved across all MFS families are assigned as x.0. This defines x.0 for TMHs 4, 5, and 10 via conserved glycine residues.
2. The conserved glycine residues in TMHs 4, 5, and 10 are also involved in conserved contacts with TMHs 2, 1, and 9, respectively (see Tables 3, 4 and Tables S2–S15). The second residue involved in these contacts (i.e., on the opposing helix) was used for numbering in TMHs 1, 2, and 9. Positions on TMHs 1, 2, and 9 are defined this way.
3. For the remaining helices, the conserved superfamily-wide contacts were used (Table 3 and Tables S2–S15). The x.0 position was defined as the most conserved residue within a contact.

**Table 4:** **Summary of proposed numbering scheme**.

**TM helix**	**How assigned**
1	Contact with TMH 5 (either polar, glycine or small hydrophobic)
2	Contact with TMH 4 (most conserved glycine)
3	Contact with TMH 6 (most conserved small hydrophobic)
4	Contact with TMH 2 (most conserved glycine)
5	Contact with TMH 1 (either polar, glycine or small hydrophobic)
6	Contact with TMH 3 (most conserved small hydrophobic)
7	Contact with TMH 11 (most conserved polar)
8	Contact with TMH 5 (most conserved polar or small hydrophobic)
9	Contact with TMH 10 (most conserved glycine or small hydrophobic if glycine not present in sequence)
10	Contact with TMH 9 (most conserved glycine)
11	Contact with TMH 7 (most conserved polar residue)
12	Contact with TMH 9 (most conserved small hydrophobic)

In TMHs 4, 5, and 10 these correspond with the conserved glycine motifs present on those helices. In other TMHs, the residues are mostly in the same spatial location on the helix, but sometimes the position changes and thus they are easier to track via their conserved contacts rather than conservation alone.

**Figure 6:**
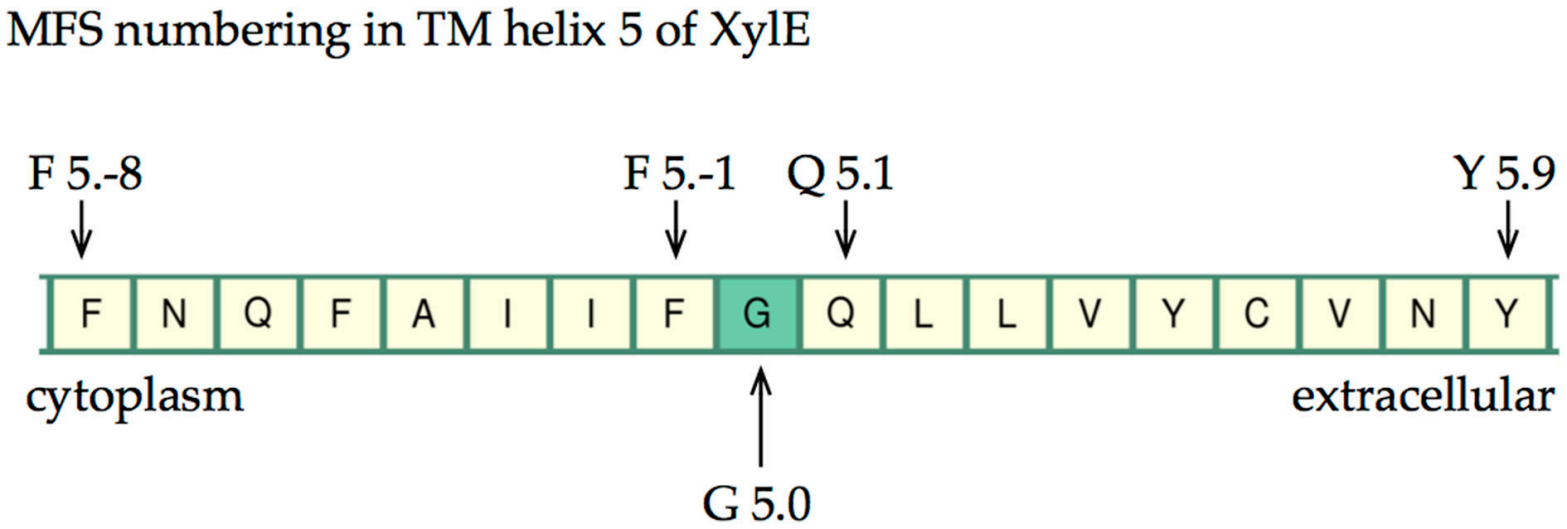
**The numbering scheme using TMH 5 of XylE as an example**. The most conserved site is labeled G5.0 and then increasing negative values are given toward the cytoplasm and increasing positive values toward the extracellular cavity.

**Figure 7:**
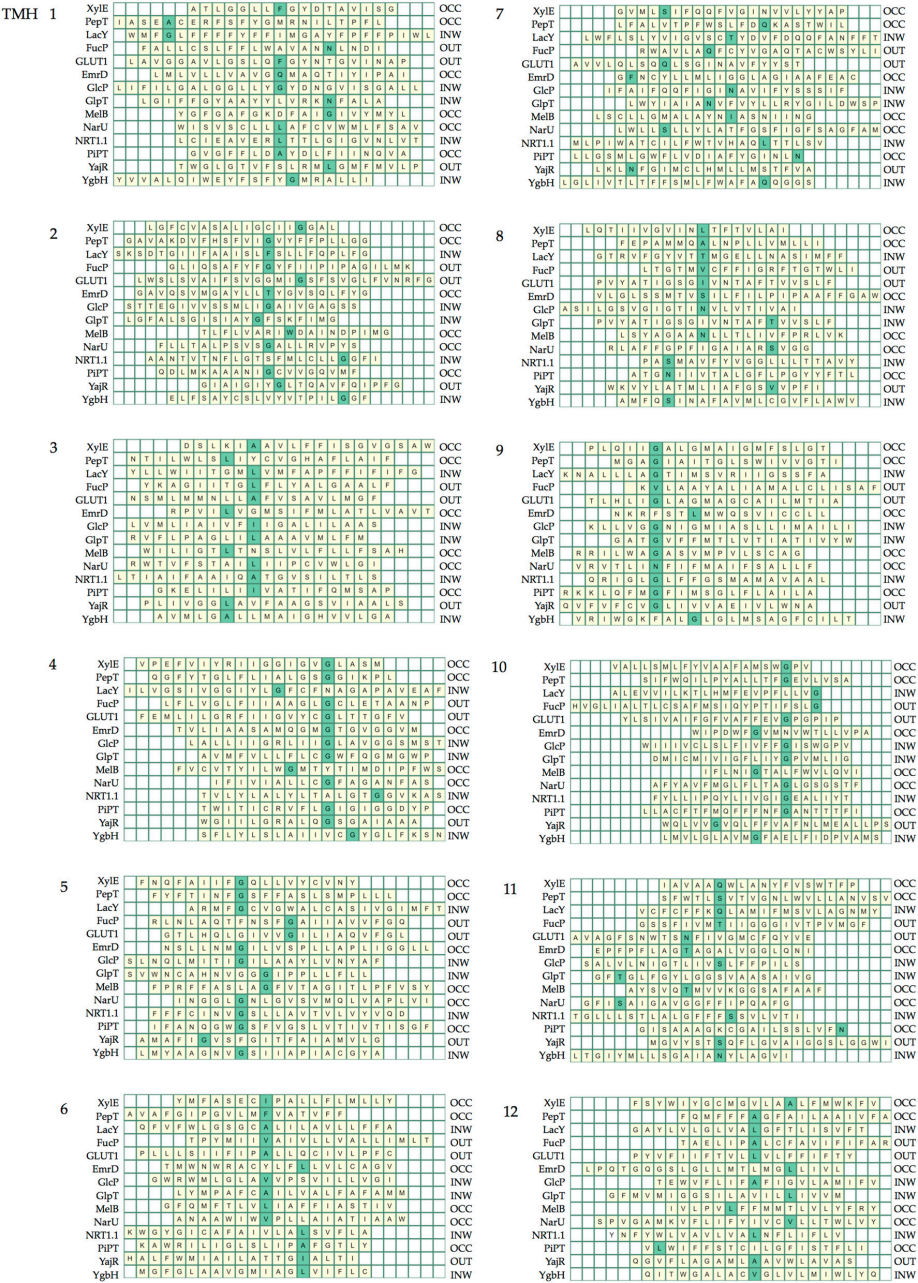
**The position of the numbered residue on each helix**. Each line of the heat maps corresponds to the helix in each crystal structure. The helix lengths are defined by those within the region of the bilayer given in the OPM database and helices are different lengths depending on the helix tilt and whether there are kinks. The helices are aligned according to the rotation of the helix such that numbered sites in the same structural position are aligned.

Table 5 summarizes the assignment. Note that the conserved contact approach actually recapitulates the identification of the conserved residues in helices 4, 5, and 10. Once x.0 is determined in a helix, the numbering will increase from 0 when moving toward the extracellular side of the TM region and will decrease from 0 when moving to the cytoplasmic side of the TM region (an example is shown in TM helix 5 of XylE in Figure 6). The majority of x.0 sites are conserved glycine residues as identified in TMHs 4, 5, and 10 or the subsequent residue involved in the conserved contact for these glycine residues in TMHs 2, 1, and 9, respectively. The conserved glycine in TMH 11 was not suitable to use for the numbering because the position moved according to the crystal structure investigated. Therefore, 11.0 corresponded to the contact between TMHs 7 and 11, which is a polar-polar contact. The numbering for all helices from all the structures considered is shown in Table 4 and illustrated in Figure 7. For each helix, the x.0 position is in structurally similar positions when compared to the orientation of helices in the X-ray crystal structures.

**Table 5:** **The numbering in MFS proteins**.

**Protein**	**H1**	**H2**	**H3**	**H4**	**H5**	**H6**	**H7**	**H8**	**H9**	**H10**	**H11**	**H12**
XylE (4JA4)	17–33	57–74	87–104	126–145	166–183	201–218	281–301	316–333	343–361	371–390	410–427	443–463
	G25	G71	A92	G141	G174	A210	S285	T329	G348	G388	Q415	A456
	**G1.0**	**G2.0**	**A3.0**	**G4.0**	**G5.0**	**A6.0**	**S7.0**	**T8.0**	**G9.0**	**G10.0**	**G11.0**	**G12.0**
PepT (2XUT)	18–39	53–75	86–105	110–129	153–172	178–195	304–324	335–352	376–393	404–424	444–464	483–500
	A22	G66	L93	G124	G160	F189	T309	A342	G379	G418	S449	A489
	**A1.0**	**G2.0**	**L3.0**	**G4.0**	**G5.0**	**F6.0**	**T7.0**	**A8.0**	**G9.0**	**G10.0**	**S11.0**	**A12.0**
LacY (1PV6)	10–35	41–64	75–96	103–128	143–163	167–187	222–248	257–278	289–309	312–332	352–373	380–399
	G13	F55	L84	G111	G147	A177	Q248	T266	G296	G332	S366	L390
	**G1.0**	**F2.0**	**L3.0**	**G4.0**	**G5.0**	**A6.0**	**Q7.0**	**T8.0**	**G9.0**	**G10.0**	**S11.0**	**L12.0**
FucP (3O7Q)	28–47	64–86	90–108	119–140	154–174	210–229	261–282	301–319	326–345	348–372	383–403	412–430
	N43	G73	L98	G132	G165	V219	Q267	V306	L328	G372	T390	A419
	**N1.0**	**G2.0**	**L3.0**	**G4.0**	**G5.0**	**V6.0**	**Q7.0**	**V8.0**	**L9.0**	**G10.0**	**T11.0**	**A12.0**
GLUT1 (4PYP)	14–36	64–91	94–112	119–140	157–176	187–206	275–295	306–326	335–354	366–387	402–426	431–449
	G27	G79	A103	G134	G167	A197	Q283	I315	G340	G382	N415	L441
	**G1.0**	**G2.0**	**A3.0**	**G4.0**	**G5.0**	**A6.0**	**Q7.0**	**I8.0**	**G9.0**	**G10.0**	**S11.0**	**L12.0**
EmrD (2GFP)	11–31	43–64	73–92	97–116	134–155	157–175	208–229	237–261	267–283	289–306	326–345	357–378
	Q21	T55	L77	G109	G140	L169	N210	S246	L279	G295	T334	L374
	**Q1.0**	**T2.0**	**L3.0**	**G4.0**	**G5.0**	**L6.0**	**N7.0**	**S8.0**	**L9.0**	**G10.0**	**T11.0**	**L12.0**
GlcP (4LDS)	7–31	41–64	74–92	95–115	134–154	159–178	244–266	275–295	305–325	335–355	376–395	400–418
	G20	G54	I83	G106	G143	V168	N256	N287	G310	G349	S388	A408
	**G1.0**	**G2.0**	**I3.0**	**G4.0**	**G5.0**	**V6.0**	**N7.0**	**N8.0**	**G9.0**	**G10.0**	**S11.0**	**A12.0**
GlpT (1PW4)	32–51	65–84	93–110	121–139	159–178	190–208	255–277	292–311	322–340	349–369	386–405	416–435
	N47	G77	L100	G131	G168	A197	N262	T306	G325	G363	T388	L431
	**N1.0**	**G2.0**	**L3.0**	**G4.0**	**G5.0**	**A6.0**	**N7.0**	**T8.0**	**G9.0**	**G10.0**	**T11.0**	**L12.0**
MelB (4M64)	11–29	46–63	79–98	107–129	147–169	178–196	233–252	272–291	295–312	332–347	368–385	415–432
	G23	W54	L91	G117	G156	L186	N244	N279	G301	G337	T373	L420
	**G1.0**	**W2.0**	**L3.0**	**G4.0**	**G5.0**	**L6.0**	**N7.0**	**N8.0**	**G9.0**	**G10.0**	**T11.0**	**L12.0**
NarU (4IU8)	37–56	73–91	101–119	130–147	167–187	211–229	254–277	290–307	316–334	347–367	405–423	431–454
	L44	G83	L110	G139	G172	V218	S258	S304	N322	G362	S408	V447
	**L1.0**	**G2.0**	**L3.0**	**G4.0**	**G5.0**	**V6.0**	**S7.0**	**S8.0**	**N9.0**	**G10.0**	**S11.0**	**V12.0**
NRT1.1 (5A2N)	38–57	70–91	100–119	147–166	193–213	218–237	342–364	381–399	421–439	462–481	500–520	542–561
	G50	G88	A110	G161	G200	L232	Q358	S383	G426	G475	S514	L554
	**G1.0**	**G2.0**	**A3.0**	**G4.0**	**G5.0**	**L6.0**	**Q7.0**	**S8.0**	**G9.0**	**G10.0**	**S11.0**	**L12.0**
PiPT (4J05)	39–56	74–92	106–123	133–151	173–195	209–227	313–333	358–377	385–404	415–436	452–469	481–499
	A46	G84	I113	G143	G180	A222	N333	N361	G392	G429	N470	L482
	**A1.0**	**G2.0**	**I3.0**	**G4.0**	**G5.0**	**A6.0**	**N7.0**	**N8.0**	**G9.0**	**G10.0**	**N11.0**	**L12.0**
YajR (3WDO)	14–33	51–69	79–98	102–119	136–155	165–183	215–234	250–268	279–298	303–325	340–361	368–386
	L26	G58	L85	G112	G141	I179	N218	V264	G286	G308	S346	A378
	**L1.0**	**G2.0**	**L3.0**	**G4.0**	**G5.0**	**I6.0**	**N7.0**	**V8.0**	**G9.0**	**G10.0**	**S11.0**	**A12.0**
YgbH (4Q65)	13–33	49–67	78–95	101–120	142–161	169–187	267–289	311–331	342–362	379–400	414–433	461–480
	G27	G65	A83	G115	G150	L182	Q285	S315	G351	G388	N427	V470
	**G1.0**	**G2.0**	**A3.0**	**G4.0**	**G5.0**	**L6.0**	**Q7.0**	**S8.0**	**G9.0**	**G10.0**	**N11.0**	**V12.0**

TM helices were taken as those predicted by the OPM database (Lomize et al., 2006). The x.0 residue of each helix is summarized in Table 4. The helix is numbered x.0, where x is the helix number and it is prefixed by the conserved residue, for example in TM helix 5, the most conserved residue is a glycine, therefore it is G5.0. Residues from this residue toward the cytoplasm are labeled as negative numbers and residues toward the periplasm are given positive numbers (for example TM helix 5 in XylE is FNQFAIIF**G**QLLVYCVNY giving: …, I5.-2, F5.-1, G5.0, Q5.1, L5.2, …). The proposed numbering start points are indicated in bold for each helix from each protein.

A couple of interesting observations arise upon further inspection of known MFS crystal structures. The first is that the conserved aromatic residue on TMH 7 of XylE (Y7.0) is closer to the periplasmic side of the helix compared with LacY, PepT, FucP, and GLUT1. This is most likely because it is not involved in a critical contact. The second is that position 1.0 in PepT and LacY, differs in location when compared to XylE, FucP, and GLUT1. This can be accounted for by the flexibility of TMH 1 in the transport mechanism that enables the contact points to change according to conformation.

From our analysis, it appears that there is less symmetry than might be expected in the contacts between the two 6-TMH domains. Whilst, the TMH 2 and 4 contact defines the numbering in domain one, the numbering of TMH 8 comes from an interaction with TMH 5. Similarly, it is a contact with TMH 10 that defines which residue is the anchor point in TMH 9, whereas in domain one, a contact between TMHs 3 and 6 defines the numbering in both those helices. Whilst this does not disagree with the inverted repeat topology of Radestock and Forrest (2011), since that investigates the conservation between whole helices rather than single residues on them, it does imply a subtlety in the conservation of contacts. It is impossible to say whether this is directly caused by evolutionary pressure, but it does pose the question of whether there is a slightly different role for each domain in the alternating access mechanism.

## Conclusions

Alignment of MFS family proteins is made difficult by their low sequence similarity, which in turn makes homology modeling difficult. The problem is compounded by the fact that many structures now exist in different states. In this work we have tried to ascertain which positions may be structurally common via contact analysis of the structures. By combining contact analysis with conservation analysis we have suggested a way to identify “anchor” points on each of the TMHs that should aid the modeling process. We have found that the majority of contacts remain static across the different conformational states. Our analysis would suggest that these contact positions would be particularly intolerant to mutation, but a systematic study would be required to fully address that. There are only small and helix specific (TMHs 1, 7, and 10 predominantly) rearrangements that take place on top of the rigid body rotation of each domain. To facilitate cross-family comparison we have also devised a numbering scheme, similar in essence to that proposed for GPCRs. We anticipate that by exploiting this analysis, homology modeling of MFS proteins should be improved.

## Availability of software

The R-script used to perform the analysis is available on request from the authors.

## Author contributions

PB and ZS designed the research. JL performed the research and developed the computational methods. JL, ZS, and PB wrote the manuscript. All authors approved the final version and accept accountability for its accuracy.

## Funding

JL is a BBSRC-funded (BB/F01709X/1) student in receipt of additional financial support from UCB BioPharma SPRL.

### Conflict of interest statement

The authors declare that the research was conducted in the absence of any commercial or financial relationships that could be construed as a potential conflict of interest. ZS is an employee of UCB BioPharma SPRL.

## Abbreviations

MFS: major facilitator superfamily.
