# Supplementary material for: A Numbering System for MFS Transporter Proteins
Source: Front Mol Biosci. 2016 Jun 2;3:21. doi: 10.3389/fmolb.2016.00021 (PMC4889909; doi:10.3389/fmolb.2016.00021)
Supplement: Supplementary file 1 [file DataSheet1.pdf]

Supporting Information: A Numbering System for MFS Transporter Proteins

Joanna Lee[1], Zara A. Sands[2] and Philip C. Biggin[1]*

[1]Department of Biochemistry, University of Oxford, South Parks Road, Oxford, OX1 3QU, United Kingdom.

[2]UCB Pharma S.A., Chemin du Foriest, B-1420 Braine-l'Alleud, Belgium.

*To whom correspondence should be addressed.
Email: philip.biggin@bioch.ox.ac.uk
Tel. +44 1865 613305
Fax. +44 1865 613238

Frontiers Molecular Biosciences

Keywords: Homology modelling, LacY, alternating access, transport.

SI FIGURE 1. The heatmap showing the glycine-based contacts between helices. The possible contacts reflect the analysis over all the different types of contact possible (see main text for details).

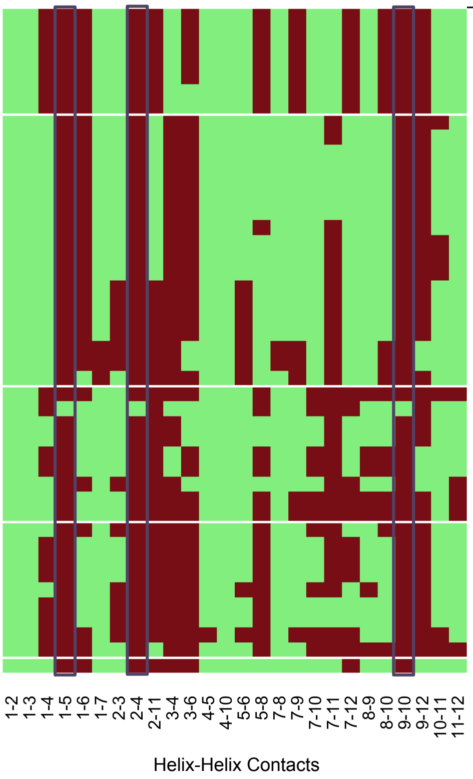

Family	Protein	PDB	Transport	State
lacy_ symp	LacY	1PV6	lactose/H^+ sym	INW, apo, C154G
		1PV7		INW, TDG bound, C154G
		2V8N		INW, WT
		2CFP		INW, apo, acidic pH
		2CFQ		INW, apo neutral pH
		4OAA		OUT OCC, TDG bound, G46W
		2Y5Y		INW, TGA bound, cys mutations
PTR	PepT	2XUT	peptide/H^+ sym	OCC, ligand density, N2T,S3T
		4LEP		INW, AFS bound
		4IKV		INW, apo
		4IKW		INW, SO_4 bound
		4IKX		INW, apo, E310Q
		4IKY		INW, SO_4 bound, E310Q
		4IKZ		INW, alafosfalin bound, E310Q
		4APS		INW, apo
		4D2B		INW, apo
		4D2C		INW, ALA-PHE bound
		4D2D		INW, tri-ALA bound
		4TPG		INW, ALA-DBY-ALA bound
		4TPH		INW, ALA-DBY-DBY bound
		4TPJ		INW, tri-ALA bound
	YgbH	4Q65		INW, apo, L472V
	NRT1.1	5A2N	nitrate/H^+ sym	INW, apo
		5A2O		INW, NO_3 bound
		4OH3		INW, NO_3 bound
MFS_1	GlpT	1PW4	G3P/Pi anti	INW, apo
	EmrD	2GFP	multidrug/H^+ anti	OCC, apo
	FucP	3O7P	fucose/H^+ sym	OUT, BNG bound, N162A
		3O7Q		OUT, BNG bound
	NarU	4IU8	nitrate/nitrite	OCC, NO_3 bound, MSE
		4IU9		OCC, apo
	YajR	3WDO	multidrug/H^+ sym	OUT, apo
	NarK	4JRE	NO_3^-/NO_2^- anti	OCC, NO_2 bound, (GYP)
		4JR9		OCC, apo, (GYP)
sugar_tr	GlcP	4LDS	glucose/H^+ sym	INW, apo
	XylE	4GBY	xylose/H^+ sym	OUT OCC, D-xylose bound
		4GBZ		OUT OCC, D-glucose bound
		4GC0		OUT OCC, 6BG bound
		4QIQ		INW, zinc-bound
		4JA3		INW OCC, D-xylose bound
		4JA4		INW OCC, apo
	GLUT1	4PYP	glucose/H^+ sym	OUT, BNG bound, N45T/E329Q
	PiPT	4J05	PO_4/H^+ sym	INW OCC, PO_4 bound
MFS_2	MelB	4M64	melibiose/Na^+	OCC, P4P bound

SI FIGURE 2. The heatmap showing contacts between helices made by small hydrophobic residues. The possible contacts reflect the analysis over all the different types of contact possible (see main text for details).

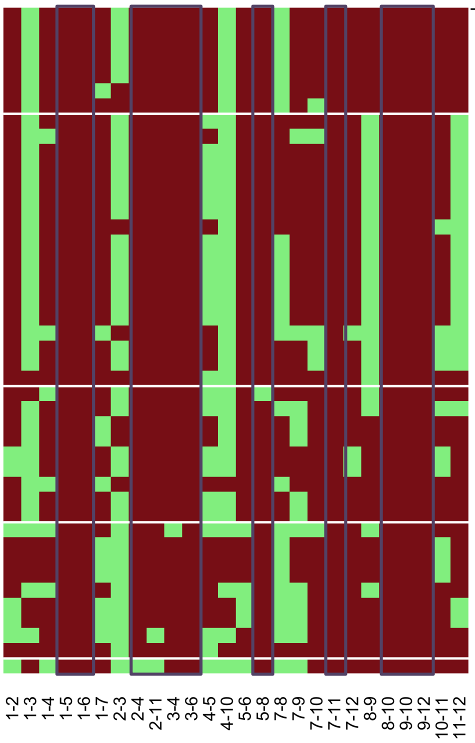

Family	Protein	PDB	Transport	State
lacy_ symp	LacY	1PV6	lactose/H^+ sym	INW, apo, C154G
		1PV7		INW, TDG bound, C154G
		2V8N		INW, WT
		2CFP		INW, apo, acidic pH
		2CFQ		INW, apo neutral pH
		4OAA		OUT OCC, TDG bound, G46W
		2Y5Y		INW, TGA bound, cys mutations
PTR	PepT	2XUT	peptide/H^+ sym	OCC, ligand density, N2T,S3T
		4LEP		INW, AFS bound
		4IKV		INW, apo
		4IKW		INW, SO_4 bound
		4IKX		INW, apo, E310Q
		4IKY		INW, SO_4 bound, E310Q
		4IKZ		INW, alafosfalin bound, E310Q
		4APS		INW, apo
		4D2B		INW, apo
		4D2C		INW, ALA-PHE bound
		4D2D		INW, tri-ALA bound
		4TPG		INW, ALA-DBY-ALA bound
		4TPH		INW, ALA-DBY-DBY bound
		4TPJ		INW, tri-ALA bound
	YgbH	4Q65		INW, apo, L472V
	NRT1.1	5A2N	nitrate/H^+ sym	INW, apo
		5A2O		INW, NO_3 bound
		4OH3		INW, NO_3 bound
MFS_1	GlpT	1PW4	G3P/Pi anti	INW, apo
	EmrD	2GFP	multidrug/H^+ anti	OCC, apo
	FucP	3O7P	fucose/H^+ sym	OUT, BNG bound, N162A
		3O7Q		OUT, BNG bound
	NarU	4IU8	nitrate/nitrite	OCC, NO_3 bound, MSE
		4IU9		OCC, apo
	YajR	3WDO	multidrug/H^+ sym	OUT, apo
	NarK	4JRE	NO_3^-/NO_2^- anti	OCC, NO_2 bound, (GYP)
		4JR9		OCC, apo, (GYP)
sugar_tr	GlcP	4LDS	glucose/H^+ sym	INW, apo
	XylE	4GBY	xylose/H^+ sym	OUT OCC, D-xylose bound
		4GBZ		OUT OCC, D-glucose bound
		4GC0		OUT OCC, 6BG bound
		4QIQ		INW, zinc-bound
		4JA3		INW OCC, D-xylose bound
		4JA4		INW OCC, apo
	GLUT1	4PYP	glucose/H^+ sym	OUT, BNG bound, N45T/E329Q
	PiPT	4J05	PO_4/H^+ sym	INW OCC, PO_4 bound
MFS_2	MelB	4M64	melibiose/Na^+	OCC, P4P bound

SI FIGURE 3. The heatmap showing contacts between helices made by large hydrophobic residues. The possible contacts reflect the analysis over all the different types of contact possible (see main text for details).

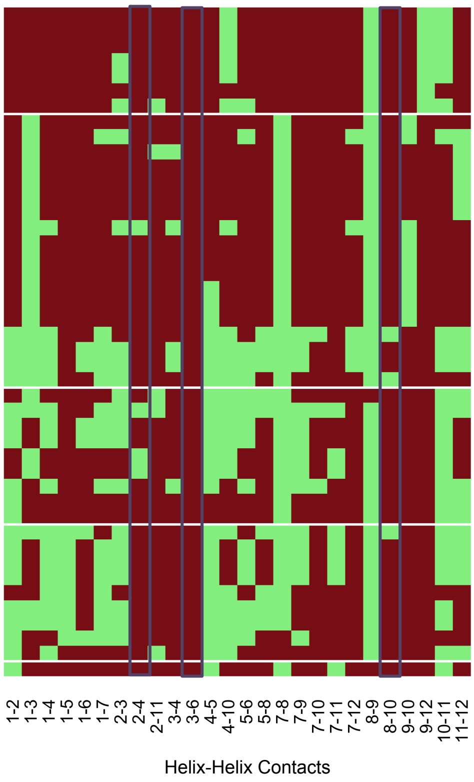

Family	Protein	PDB	Transport	State
lacy_ symp	LacY	1PV6	lactose/H^+ sym	INW, apo, C154G
		1PV7		INW, TDG bound, C154G
		2V8N		INW, WT
		2CFP		INW, apo, acidic pH
		2CFQ		INW, apo neutral pH
		4OAA		OUT OCC, TDG bound, G46W
		2Y5Y		INW, TGA bound, cys mutations
PTR	PepT	2XUT	peptide/H^+ sym	OCC, ligand density, N2T,S3T
		4LEP		INW, AFS bound
		4IKV		INW, apo
		4IKW		INW, SO_4 bound
		4IKX		INW, apo, E310Q
		4IKY		INW, SO_4 bound, E310Q
		4IKZ		INW, alafosfalin bound, E310Q
		4APS		INW, apo
		4D2B		INW, apo
		4D2C		INW, ALA-PHE bound
		4D2D		INW, tri-ALA bound
		4TPG		INW, ALA-DBY-ALA bound
		4TPH		INW, ALA-DBY-DBY bound
		4TPJ		INW, tri-ALA bound
	YgbH	4Q65		INW, apo, L472V
	NRT1.1	5A2N	nitrate/H^+ sym	INW, apo
		5A2O		INW, NO_3 bound
		4OH3		INW, NO_3 bound
MFS_1	GlpT	1PW4	G3P/Pi anti	INW, apo
	EmrD	2GFP	multidrug/H^+ anti	OCC, apo
	FucP	3O7P	fucose/H^+ sym	OUT, BNG bound, N162A
		3O7Q		OUT, BNG bound
	NarU	4IU8	nitrate/nitrite	OCC, NO_3 bound, MSE
		4IU9		OCC, apo
	YajR	3WDO	multidrug/H^+ sym	OUT, apo
	NarK	4JRE	NO_3^-/NO_2^- anti	OCC, NO_2 bound, (GYP)
		4JR9		OCC, apo, (GYP)
sugar_tr	GlcP	4LDS	glucose/H^+ sym	INW, apo
	XylE	4GBY	xylose/H^+ sym	OUT OCC, D-xylose bound
		4GBZ		OUT OCC, D-glucose bound
		4GC0		OUT OCC, 6BG bound
		4QIQ		INW, zinc-bound
		4JA3		INW OCC, D-xylose bound
		4JA4		INW OCC, apo
	GLUT1	4PYP	glucose/H^+ sym	OUT, BNG bound, N45T/E329Q
	PiPT	4J05	PO_4/H^+ sym	INW OCC, PO_4 bound
MFS_2	MelB	4M64	melibiose/Na^+	OCC, P4P bound

SI Table 1. The numbering scheme used in Class A GPCRs. The table is taken from Ballesteros and Weinstein [1]. The most conserved residue on each helix is numbered x.50 and then each residue of the helix is numbered up or down from 50. (AA = amino acid, βγADR is the GPCR protein, β_2-adrenergic receptor).

TMH	Conserved residue	Amino acid identifier	Amino acid in β_2 ADR	Amino acid identifier in β_2 ADR
1	Asn	N 1.50	51	N 1.50 (51)
2	Asp	D 2.50	79	D 2.50 (79)
3	Arg	R 3.50	131	R 3.50 (131)
4	Trp	W 4.50	158	W 4.50 (158)
5	Pro	P 5.50	211	P 5.50 (211)
6	Pro	P 6.50	288	P 6.50 (288)
7	Pro	P 7.50	323	P 7.50 (323)

SI Table 2. The position of the conserved contacts used in the numbering in XylE.

Helix	Conserved Residue	Conserved Contact	Contact Distance (Å)	Residues in Contact	Residue to Number	Helix Range
1	-	1-5 Gly	CA - CA: 4.9	G25 - G174	G25	17-33
2	-	2-4 Gly	CA-CA: 4.6	G71 - G141	G71	57-74
3	-	3-6 small	CA - CA: 5.6	A92 - A210	A92	87-104
4	Gly	2-4 Gly	CA-CA: 4.6	G71 - G141	G141	126-145
5	Gly	1-5 Gly	CA - CA: 4.9	G25 - G174	G174	166-183
6	-	3-6 small	CA - CA: 5.6	A92 - A210	A210	201-218
7	aromatic	7-11 STNQ	OG - CA: 3.8	S285 - Q415	S285	281-301
8	-	5-8 small	CD1 - O: 5.1	Q168 - A326	A326	316-333
9	-	9-10 Gly	CA - CA: 5.8	G348 - A382	G348	343-361
10	Gly	9-10 Gly	CA - CA: 5.8	G348 - A382	G388	371-390
11	Gly	7-11 STNQ	OG - CA: 3.8	S285 - Q415	Q415	410-427
12	-	9-12 small	CA - CA: 4.7	I346 - A456	A456	443-463

SI Table 3. The position of the conserved contacts used in the numbering in PepT.

Helix	Conserved Residue	Conserved Contact	Contact Distance (Å)	Residues in Contact	Residue to Number	Helix Range
1	-	1-5 Gly	C - CA: 6.9	G160 - A/G22	A22	18-39
2	-	2-4 Gly	CA - CA: 6.7	G124 - G66	G66	53-75
3	-	3-6 large	CA - CA: 8.3	L93 - F189	L93	86-105
4	Gly	2-4 Gly	CA - CA: 6.7	G124 - G66	G124	110-129
5	Gly	1-5 Gly	C - CA: 6.9	G160 - A/G22	G160	153-172
6	-	3-6 large	CA - CA: 8.3	L93 - F189	F189	178-195
7	aromatic	7-11 STNQ	CA - O: 4.2	T309 - S449	T309	304-324
8	-	5-8 STNQ	CA - O: 4.5	S165 - A342	A342	335-352
9	-	9-10 Gly	CA - CA: 4.9	G379 - G418	G379	376-393
10	Gly	9-10 Gly	CA - CA: 4.9	G379 - G418	G418	404-424
11	-	7-11 STNQ	CA - O: 4.2	T309 - S449	S449	444-464
12	-	9-12 small	CA - CA: 5.2	I388 - A489	A489	483-500

SI Table 4. The position of the conserved contacts used in the numbering in LacY.

Helix	Conserved Residue	Conserved Contact	Contact Distance (Å)	Residues in Contact	Residue to Number	Helix Range
1	-	1-5 Gly	CA - CA: 6.0	G147 - G13	G13	10-35
2	-	2-4 large	C - CD2: 6.7	G111 - F55	F55	41-64
3	-	3-6 small	C - C: 6.7	L84 - A177	L84	75-95
4	-	2-4 large	C - CD2: 6.7	G111 - F55	G111	103-128
5	Gly	1-5 Gly	CA - CA: 6.0	G147 - G13	G147	143-163
6	-	3-6 small	C - C: 6.7	L84 - A177	A177	167-187
7	aromatic	7-11 STNQ	NE2 - CA: 4.4	Q241 - S366	Q241	222-248
8	-	5-8 STNQ	CA - CA: 6.2	S156 - T266	T266	257-278
9	-	9-10 Gly	CA - CA: 7.9	G296 - L329	G296	289-309
10	Gly	9-10 Gly	CA - CA: 7.9	G296 - L329	G332	312-332
11	Gly	7-11 STNQ	NE2 - CA: 4.4	Q241 - S366	S366	352-373
12	-	9-12 small	CA - CD1: 5.6	I304 - L390	L390	380-399

SI Table 5. The position of the conserved contacts used in the numbering in FucP.

Helix	Conserved Residue	Conserved Contact	Contact Distance (Å)	Residues in Contact	Residue to Number	Helix Range
1	-	1-2 STNQ	CG - CA: 6.9	N43 - G165	N43	28-47
2	-	2-4 Gly	CA - O: 2.9	G73 - G132	G73	64-86
3	-	3-6 large	CG - CG1: 7.7	L98 - V219	L98	90-108
4	Gly	2-4 Gly	CA - O: 2.9	G73 - G132	G132	119-140
5	Gly	1-5 Gly	CG - CA: 6.9	N43 - G165	G165	154-174
6	-	3-6 small	CG - CG1: 7.7	L98 - V219	V219	210-229
7	aromatic	7-11 STNQ	CA - CB: 5.7	Q267 - T390	Q267	261-282
8	-	8-10 small	CD1 - CA: 5.0	I167 - V306	V306	301-319
9	-	9-10 small	CG2 - CA: 4.7	I363 - L328	L328	326-345
10	Gly	9-10 Gly	CG2 - CA: 4.7	I363 - L328	G372	348-372
11	Gly	7-11 STNQ	CA - CB: 5.7	Q267 - T390	T390	383-403
12	-	9-12 small	CB - CA: 5.0	L340 - A419	A419	412-430

SI Table 6. The position of the conserved contacts used in the numbering in GLUT1.

Helix	Conserved Residue	Conserved Contact	Contact Distance (Å)	Residues in Contact	Residue to Number	Helix Range
1	-	1-5 Gly	CA - CA: 3.6	G27 - G167	G27	14-36
2	-	2-4 Gly	CA - CA: 3.8	G79 - G134	G79	64-91
3	-	3-6 large	CB - CB: 4.1	A103 - A197	A103	94-112
4	Gly	2-4 Gly	CA - CA: 3.8	G79 - G134	G134	119-140
5	Gly	1-5 Gly	CA - CA: 3.6	G27 - G167	G167	157-176
6	-	3-6 small	CB - CB: 4.1	A103 - A197	A197	187-206
7	aromatic	7-11 STNQ	CA - CB: 7.5	Q283 - N415	Q283	275-295
8	-	5-8 small	CD1 - CB: 4.2	L169 - I315	I315	306-326
9	-	9-10 Gly	CA - CA: 3.8	G340 - G382	G340	335-354
10	Gly	9-10 Gly	CA - CA: 3.8	G340 - G382	G382	366-387
11	Gly	7-11 STNQ	CA - CB: 7.5	Q283 - N415	N415	402-426
12	-	9-12 small	CB - CB: 3.7	A345 - L441	L441	431-449

SI Table 7. The position of the conserved contacts used in the numbering in EmrD.

Helix	Conserved Residue	Conserved Contact	Contact Distance (Å)	Residues in Contact	Residue to Number	Helix Range
1	-	1-5 Gly	OE1 - N: 5.2	Q21 - G140	Q21	11-31
2	-	2-4 Gly	CA - CA: 6.6	T55 - G109	T55	43-64
3	-	3-6 small	O - CD2: 3.3	L77 - L169	L77	73-92
4	Gly	2-4 Gly	CA - CA: 6.6	T55 - G109	G109	97-116
5	Gly	1-5 Gly	OE1 - N: 5.2	Q21 - G140	G140	134-155
6	-	3-6 small	O - CD2: 3.3	L77 - L169	L169	157-175
7	aromatic	7-11 STNQ	OD1 - OG1: 7.0	N210 - T334	N210	208-229
8	-	5-8 STNQ	CB - C: 3.9	S144 - S246	S246	237-261
9	-	9-10 Gly	CD1 - CA: 3.4	L279 - G295	L279	267-283
10	Gly	9-10 Gly	CD1 - CA: 3.4	L279 - G295	G295	289-306
11	Gly	7-11 STNQ	OD1 - OG1: 7.0	N210 - T334	T334	326-345
12	-	9-12 small	CD1 - CB: 5.0	L273 - L374	L374	357-378

SI Table 8. The position of the conserved contacts used in the numbering in GlcP.

Helix	Conserved Residue	Conserved Contact	Contact Distance (Å)	Residues in Contact	Residue to Number	Helix Range
1	-	1-5 Gly	C - C: 4.5	G20 - G143	G20	7-31
2	-	2-4 Gly	CA - C: 4.1	G54 - G106	G54	41-62
3	-	3-6 small	CG1 - CB: 5.2	I83 - V168	I83	74-92
4	Gly	2-4 Gly	CA - C: 4.1	G54 - G106	G106	95-115
5	Gly	1-5 Gly	C - C: 4.5	G20 - G143	G143	134-154
6	-	3-6 small	CG1 - CB: 5.2	I83 - V168	V168	159-178
7	aromatic	7-11 STNQ	O - C: 7.1	N256 - S388	N256	244-266
8	-	5-8 STNQ	OG1 - ND2: 3.1	T141 - N287	N287	275-295
9	-	9-10 Gly			G310	305-325
10	Gly	9-10 Gly			G349	335-355
11	Gly	7-11 STNQ	O - C: 7.1	N256 - S388	S388	376-395
12	-	9-12 small	CD1 - N: 3.3	L318 - A408	A408	400-418

SI Table 9. The position of the conserved contacts used in the numbering in GlpT.

Helix	Conserved Residue	Conserved Contact	Contact Distance (Å)	Residues in Contact	Residue to Number	Helix Range
1	-	1-5 Gly	OD1 - CB: 4.1	N47 - G168	N47	32-51
2	-	2-4 Gly	N - C: 6.4	G77 - G131	G77	65-84
3	-	3-6 small	CB - CD2: 4.0	L100 - A197	L100	93-110
4	Gly	2-4 Gly	N - C: 6.4	G77 - G131	G131	121-139
5	Gly	1-5 Gly	OD1 - CB: 4.1	N47 - G168	G168	159-178
6	-	3-6 small	CB - CD2: 4.0	L100 - A197	A197	190-208
7	aromatic	7-11 STNQ	OD1 - CA: 4.9	N262 - T388	N262	255-277
8	-	5-8 STNQ	O - CG2: 4.9	N162 - T306	T306	292-311
9	-	9-10 Gly			G325	322-340
10	Gly	9-10 Gly			G363	349-369
		7-11 STNQ	OD1 - CA: 4.9	N262 - T388	T388	386-405
12	-	9-12 small	CG2 - CG: 4.6	V326 - L431	L431	416-435

SI Table 10. The position of the conserved contacts used in the numbering in MelB.

Helix	Conserved Residue	Conserved Contact	Contact Distance (Å)	Residues in Contact	Residue to Number	Helix Range
1	-	1-5 Gly	CA - CA: 3.8	G23 - G156	G23	11-29
2	-	2-4 large	CE3 - CA: 5.0	W54 - G117	W54	46-63
3	-	3-6 small	CD1 - CD2: 3.4	L91 - L186	L91	79-98
4	Gly	2-4 Gly	CE3 - CA: 5.0	W54 - G117	G117	107-129
5	Gly	1-5 Gly	CA - CA: 3.8	G23 - G156	G156	147-169
6	-	3-6 small	CD1 - CD2: 3.4	L91 - L186	L186	178-196
7	-	7-11 STNQ	ND2 - O: 5.1	N244 - T373	N244	233-252
8	-	5-8 STNQ	OG - N: 6.3	S153 - N279	N279	272-291
9	-	9-10 Gly	O - O: 7.6	G301 - G337	G301	295-312
10	Gly	9-10 Gly	O - O: 7.6	G301 - G337	G337	332-347
11	Gly	7-11 STNQ	ND2 - O: 5.1	N244 - T373	T373	368-385
12	-	9-12 small	CG2 - CG2: 7.3	V307 - L420	L420	415-432

SI Table 11. The position of the conserved contacts used in the numbering in NarU.

Helix	Conserved Residue	Conserved Contact	Contact Distance (Å)	Residues in Contact	Residue to Number	Helix Range
1	-	1-5 Gly	CB - CA: 4.5	L44 - G172	L44	37-56
2	-	2-4 Gly	CA - O: 3.0	G83 - G139	G83	73 - 91
3	-	3-6 small	C - CA: 7.0	L110 - V218	L110	101-119
4	Gly	2-4 Gly	CA - O: 3.0	G83 - G139	G139	130-147
5	Gly	1-5 Gly	CB - CA: 4.5	L44 - G172	G172	167-187
6	-	1-6 small	C - CA: 7.0	L110 - V218	V218	211-229
7	aromatic	7-11 STNQ	CB - N: 6.8	S258 - S408	S258	254-277
8	-	5-8 STNQ	CB - CB: 7.8	N173 - S304	S304	290-307
9	-	9-10 Gly	OD1 - N: 3.7	N322 - G362	N322	316-334
10	Gly	9-10 Gly	OD1 - N: 3.7	N322 - G362	G362	347-367
11	Gly	7-11 STNQ	CB - N: 6.8	S258 - S408	S408	405-423
12	-	9-12 small	CD1 - CG1: 3.5	I324 - V447	V447	431-454

SI Table 12. The position of the conserved contacts used in the numbering in NRT1.1.

Helix	Conserved Residue	Conserved Contact	Contact Distance (Å)	Residues in Contact	Residue to Number	Helix Range
1	-	1-5 Gly	CA - O: 7.9	G50 - G200	G50	38-57
2	-	2-4 Gly	N - CA: 6.2	G88 - G161	G88	70-91
3	-	3-6 small	CB - CD1: 5.5	A110 - L232	A110	100-119
4	Gly	2-4 Gly	N - CA: 6.2	G88 - G161	G161	147-166
5	Gly	1-5 Gly	CA - O: 7.9	G50 - G200	G200	193-213
6	-	3-6 small	CB - CD1: 5.5	A110 - L232	L232	218-237
7	aromatic	7-11 STNQ	NE2 - O: 5.1	Q358 - S514	Q358	342-364
8	-	5-8 STNQ	CA - OG: 4.2	T206 - S383	S383	381-399
9	-	9-10 Gly	CA - CA: 3.8	G426 - G475	G426	421-439
10	Gly	9-10 Gly	CA - CA: 3.8	G426 - G475	G475	462-481
11	Gly	2-11 Gly	NE2 - O: 5.1	Q358 - S514	S514	500-520
12	-	9-12 small	C - CD1: 3.9	L427 - L554	L554	542-561

SI Table 13. The position of the conserved contacts used in the numbering in PiPT.

Helix	Conserved Residue	Conserved Contact	Contact Distance (Å)	Residues in Contact	Residue to Number	Helix Range
1	-	1-5 small	CA - CA: 4.9	A46 - G180	A46	39-56
2	-	2-4 Gly	CA - CA: 3.9	G84 - G143	G84	74-92
3	-	3-6 small	CD1 - C: 4.5	I113 - A222	I113	106-123
4	Gly	2-4 Gly	CA - CA: 3.9	G84 - G143	G143	133-151
5	Gly	1-5 Gly	CA - CA: 4.9	A46 - G180	G180	173-195
6	-	3-6 small	CD1 - C: 4.5	I113 - A222	A222	209-227
7	aromatic	7-11 STNQ	OD1 - ND1: 2.9	N333 - N470	N333	313-333
8	-	5-8 STNQ	O - C: 6.8	N185 - N361	N361	358-377
9	-	9-10 Gly	CA - CA: 3.9	G392 - G429	G392	385-404
10	Gly	9-10 Gly	CA - CA: 3.9	G392 - G429	G429	415-436
11	Gly	7-11 STNQ	OD1 - ND1: 2.9	N333 - N470	N470	452-469
12	-	9-12 small	CB - CB: 3.6	A404 - L482	L482	481-499

SI Table 14. The position of the conserved contacts used in the numbering in YajR.

Helix	Conserved Residue	Conserved Contact	Contact Distance (Å)	Residues in Contact	Residue to Number	Helix Range
1	-	1-5 small	CD2 - CB: 3.5	L26 - A151	L26	14-33
2	-	2-4 Gly	N - CA: 5.5	G58 - G112	G58	51-69
3	-	3-6 small	CG - CD1: 5.4	L85 - I179	L85	79-98
4	Gly	2-4 Gly	N - CA: 5.5	G58 - G112	G112	102-119
5	Gly	1-5 Gly			G141	136-155
6	-	3-6 small	CG - CD1: 5.4	L85 - I179	I179	165-183
7	aromatic	7-11 STNQ	CA - OG: 5.1	N218 - S346	N218	215-234
8	-	5-8 small	CG2 - CG1: 4.7	V142 - V264	V264	250-268
9	-	9-10 Gly			G286	279-298
10	Gly	9-10 Gly			G308	303-325
11	Gly	2-11 Gly	CA - OG: 5.1	N219 - S346	S346	340-361
12	-	9-12 small	CG2 - CB: 3.5	V289 - A378	A378	368-386

SI Table 15. The position of the conserved contacts used in the numbering in YgbH.

Helix	Conserved Residue	Conserved Contact	Contact Distance (Å)	Residues in Contact	Residue to Number	Helix Range
1	-	1-5 Gly	N - O: 6.3	G27 - G150	G27	13-33
2	-	2-4 Gly	CA - C: 4.2	G65 - G115	G65	49-67
3	-	3-6 small	CB - CA: 5.4	A83 - L182	A83	78-95
4	Gly	2-4 Gly	CA - C: 4.2	G65 - G115	G115	101-120
5	Gly	1-5 Gly	N - O: 6.3	G27 - G150	G150	142-161
6	-	3-6 small	CB - CA: 5.4	A83 - L182	L182	169-187
7	-	7-11 STNQ	OE1 - ND2: 3.4	Q285 - N427	Q285	267-289
8	-	5-8 STNQ	CB - CB: 8.0	S151 - S315	S315	311-331
9	-	9-10 Gly	CA - C: 8.5	G351 - G388	G351	342-363
10	Gly	9-10 Gly	CA - C: 8.5	G351 - G388	G388	379-400
11	-	7-11 STNQ	OE1 - ND2: 3.4	Q285 - N427	N427	414-433
12	-	9-12 small	O - CG1: 3.3	A349 - V470	V470	461-480

References

1. Ballesteros JA, Weinstein H: Integrated methods for construction three dimensional models and computational probing of structure-function relations in G protein-coupled receptors. Methods Neurosci 1995, 25:366-428.
